# Retraction Note: LncRNA PCGEM1 induces proliferation and migration in non-small cell lung cancer cells through modulating the miR-590-3p/SOX11 axis

**DOI:** 10.1186/s12890-022-02162-0

**Published:** 2022-10-18

**Authors:** Huanshun Wen, Hongxiang Feng, Qianli Ma, Chaoyang Liang

**Affiliations:** Department of Thoracic Surgery, Friendship Hospital, No. 2 Yinghua East Street, Chaoyang District, 100029 Beijing, China

## BMC Pulmonary Medicine (2021) 21:234

10.1186/s12890-021-01600-9.

The authors have retracted this article because of problems with Fig. 1, namely:

Figure 1G (panel H1299 vs. sh-PCGEM1#2) overlaps with Fig. 5D (panel Invasion vs. sh-PCGEM1#1 + pcDNA3.1/SOX11).

Figure 1F (panel A549 vs. sh-NC) overlaps with Fig. 4 H (panel H1975 vs. pcDNA3.1-NC) of a different article [1].

The authors have stated that some of the cell biology experiments were performed by external companies. All authors agree with this retraction.

1. Kang, H., Ma, D., Zhang, J. et al. Long non-coding RNA GATA6-AS1 upregulates GATA6 to regulate the biological behaviors of lung adenocarcinoma cells. BMC Pulm Med 21, 166 (2021).
